# Sense of agency and decision making

**DOI:** 10.3389/fpsyg.2026.1816045

**Published:** 2026-05-15

**Authors:** F. Gregory Ashby

**Affiliations:** Department of Psychological & Brain Sciences, University of California, Santa Barbara, Santa Barbara, CA, United States

**Keywords:** agency, decision making, dopamine, prospect theory, risk taking, temporal discounting, utility theory

## Abstract

Agency is the sense that one has control over one's own actions and the consequences of those actions. A recent theory proposes that increases in agency disinhibit the dopamine system and thereby increase the number of tonically active dopamine neurons in the ventral tegmental area. The theory, called ADDS (Agency Disinhibits the Dopamine System), proposes a specific neural network that mediates these effects. ADDS successfully accounts for a variety of relevant neuroscience and behavioral results. This article extends ADDS to decision making by deriving many novel predictions about how the sense of agency affects many different components of the decision-making process. Specifically, it is shown that ADDS predicts that increases in agency should: 1) increase the value of positive outcomes but have relatively little effect on the value of negative outcomes; 2) increase risk taking; and 3) reduce temporal discounting, except in cases where endogenous DA levels are unusually high, in which case increases in agency should increase temporal discounting. More empirical work is needed to test these predictions rigorously. Even so, considerable existing evidence is reviewed that supports each of these predictions.

## 1 Introduction

Agency is the sense that one has control over one's own actions and the consequences that result from those actions (Moore, 2016). An enormous literature suggests that agency strongly affects many human behaviors (e.g., Bandura, 2006; Mele, 2003; Russell, 2013) and that it is a critical component of psychological health (e.g., Bandura, 1977).

Whereas most research on the sense of agency has focused on it psychological basis and its behavioral effects, there is nevertheless growing interest in its neural basis (e.g., Haggard, 2017). For example, a recent neuropsychological theory was the first to describe the neural consequences that occur when agency increases or decreases. Specifically, Ashby et al. (2024) proposed that increases in agency disinhibit the dopamine (DA) system and thereby increase the number of tonically active DA neurons in the ventral tegmental area (VTA). The theory, called ADDS (Agency Disinhibits the Dopamine System), provides a description of the specific neural network that mediates these effects and it accurately predicts a variety of relevant neuroscience and behavioral results. This article extends ADDS to decision making by deriving novel predictions about how sense of agency affects many aspects of the decision-making process, with a focus on how agency affects (1) the value or utility assigned to the various possible outcomes of a decision; (2) the willingness to accept risk; (3) subjective probability; and (4) the temporal discounting of future rewards.

## 2 The ADDS theory of how agency affects brain dopamine levels

**Figure 1:**
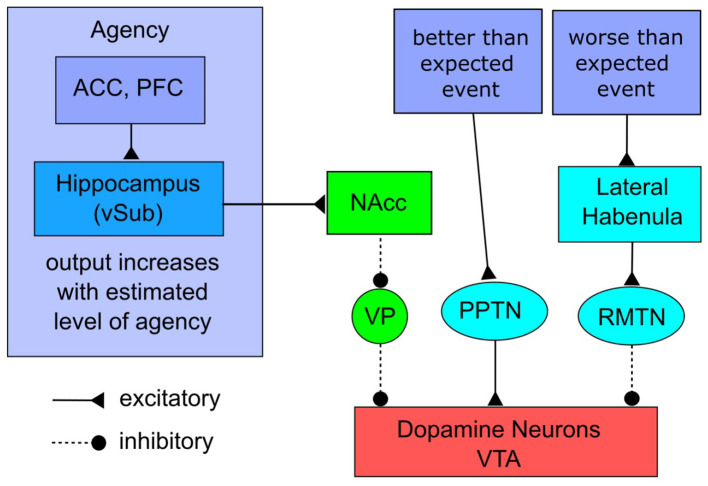
The ADDS theory of how agency modulates the firing of DA neurons. ACC, anterior cingulate cortex; PFC, prefrontal cortex; vSub, ventral subiculum; NAcc, nucleus accumbens; VP, ventral pallidum; PPTN, pedunculopontine tegmental nucleus; RMTN, rostromedial tegmental nucleus; VTA, ventral tegmental area. Adapted from Inglis et al. (2021).

The neural network that underlies the ADDS model is described in Figure 1. A rigorous justification and more detailed description of the model can be found in Ashby et al. (2024), along with a description of many empirical tests of the model's predictions. ADDS is not a theory of agency, but rather a theory of how changes in the sense of agency affect the brain, and more specifically, DA neurons in the VTA. In fact, we know that people continuously monitor their own agency, and are quick to note, for example, when environmental conditions change in a way that either increases or decreases their agency (Metcalfe and Greene, 2007). As a result, there must be some network that continuously updates estimates of agency. Because ADDS is not a theory of agency, however, it makes few assumptions about this network, or even about the exact nature of agency. As mentioned, a simple definition is that agency is the sense that one has control over one's own actions and the consequences that result from those actions (Moore, 2016). Even so, there are many different ways that “control” could be interpreted. For example, Skinner (1996) lists well over 30 different control-related terms that have appeared in the literature. ADDS makes no assumptions about exactly which of these terms best describes the inputs that are driving the Figure 1 network. As described in detail by Ashby et al. (2024), ADDS was developed primarily from the neuroscience literature on feedback contingency, which they justified by appealing to a prominent theory that assumes a strong sense of agency requires that outcomes are consistent with expectations (Wegner and Wheatley, 1999). So ADDS assumes that increases in agency, and therefore increases in the strength of the input to the Figure 1 network, increase with the accuracy of an agent's predictions about how the environment will respond to any given behavior, or in how strongly the agent believes that the environment will respond to their actions as they predict. In the terminology of Skinner (1996), agency as assumed by ADDS is therefore a form of subjective control that includes agent-ends relations.

The right half of the Figure 1 network instantiates a standard model of how positive and negative events affect the firing of DA neurons in the VTA. Considerable evidence shows that an unexpected positive event, such as a better-than-expected reward, causes reward-sensitive neurons in regions such as medial prefrontal and orbitofrontal cortices to provide excitatory inputs to the pedunculopontine tegmental nucleus, which stimulates the DA neurons in the VTA and causes the tonically active VTA DA neurons to fire phasic bursts (Kobayashi and Okada, 2007; Okada and Kobayashi, 2013; Pan and Hyland, 2005). This phasic firing raises DA levels in all VTA target regions. In contrast, if an expected positive event fails to occur—that is, if an outcome is worse than expected—then other reward-sensitive neurons stimulate the lateral habenula, which stimulates the rostromedial tegmental nucleus, which inhibits the tonically active VTA DA neurons, thereby lowering DA levels in all VTA targets (Tian and Uchida, 2015; Hong et al., 2011; Matsumoto and Hikosaka, 2007, 2009).

The left half of Figure 1, which is the more novel portion of the model, describes how changes in the sense of agency affect DA neurons in the VTA. Evidence suggests that the neural network that estimates agency includes a diverse set of cortical and subcortical regions (Haggard, 2017; Seghezzi et al., 2019). ADDS makes few specific predictions about this network, except that outputs of this network gate the amount of DA release in the VTA via projections through the ventral subiculum of the hippocampus (vSub; via projections proposed by Grace et al., 2007). See Ashby et al. (2024) for a detailed justification of this assumption.

The projections from vSub to the nucleus accumbens are excitatory and the projections from the accumbens to the ventral pallidum and from the ventral pallidum to the DA neurons of the VTA are inhibitory. Even so, a key feature of this neuroanatomy is that the tonic firing rate of ventral pallidal neurons is much higher than the tonic firing rate of nucleus accumbens neurons. Specifically, the tonic firing rate of medium spiny neurons in the nucleus accumbens averages one spike every two seconds, or less (Fabbricatore et al., 2009; Vachez et al., 2021). In contrast, Root et al. (2012) reported that the average firing rate of ventral pallidal projection neurons is 6.5 Hz, and that some of these neurons have a tonic rate as high as 71.9 Hz. Similar results were reported by Richard et al. (2016). As a result of this difference, many DA neurons in VTA are silent due to tonic inhibition by the ventral pallidum. Estimates suggest that because of this inhibition, only about half of VTA DA neurons are spontaneously active under control conditions, and these tonically firing neurons are the only ones available to respond to salient environmental events (Grace et al., 2007; Lodge and Grace, 2006). In other words, at any point in time, VTA DA neurons are in one of three states. Some are silent because of tonic inhibition from the ventral pallidum, in which case they contribute no DA to any target areas and are also unable to respond to any excitatory inputs. Other DA neurons are firing tonically, thereby contributing to a steady influx of DA to all VTA target regions (e.g., frontal cortex). Finally, a third possibility is that a VTA DA neuron that previously was firing tonically is now firing phasically in response to an excitatory input. Even a short phasic burst raises DA levels in frontal cortex above baseline for a period of 20–30 min (e.g., Feenstra and Botterblom, 1996).

ADDS predicts that when agency is high, vSub excites the nucleus accumbens, which inhibits the ventral pallidum. This releases VTA DA neurons from tonic inhibition, thereby reducing the number that are silent. The subsequent increase in the number of tonically active DA raises tonic DA levels in all VTA target brain regions and enlarges the pool of DA neurons that can respond to environmental events (i.e., fire phasically). In contrast, if agency suddenly drops, ADDS predicts that the vSub excitation of the nucleus accumbens will decrease, which reduces inhibition on the ventral pallidum, and that the resulting subsequent increase in pallidal activity will increase inhibition of the VTA DA neurons, thereby reducing the number that are tonically active.

ADDS makes two fundamental and novel predictions about the neural consequences of a change in the sense of agency. Specifically, the theory predicts that increases in agency should: (1) increase tonic DA levels in all VTA target regions (e.g., frontal cortex); and (2) increase the number of DA neurons available to respond to environmental events, which will amplify the DA response to positive and negative outcomes. Specifically, an increase in agency will increase the phasic DA response to the same better-than-expected outcome and cause a greater decrease in the DA response to any worse-than-expected outcome. Furthermore, these predictions are causal in the sense that ADDS predicts that any increase in agency, no matter what the cause, will lead to these predicted DA effects.

Inglis et al. (2021) showed that the network illustrated in Figure 1 provides a good account of a variety of different neuroscience data sets. To show this, they built a computational model of the Figure 1 network that was constructed from mathematical models of spiking neurons. The key nucleus accumbens and ventral pallidum regions were modeled as follows. The nucleus accumbens was modeled via 100 Izhikevich medium spiny neurons with a tonic firing rate of 0 Hz (Izhikevich, 2007). This model displays up- and down-states that characterize medium spiny neurons, and accurately reproduces spike trains from real medium spiny neurons (Izhikevich, 2007). The ventral pallidum was modeled by 100 quadratic integrate-and-fire neurons with a tonic firing rate of 7 Hz. Thus, the model faithfully captured the differences in the tonic firing rates of projection neurons in the nucleus accumbens and ventral pallidum.

**Figure 2:**
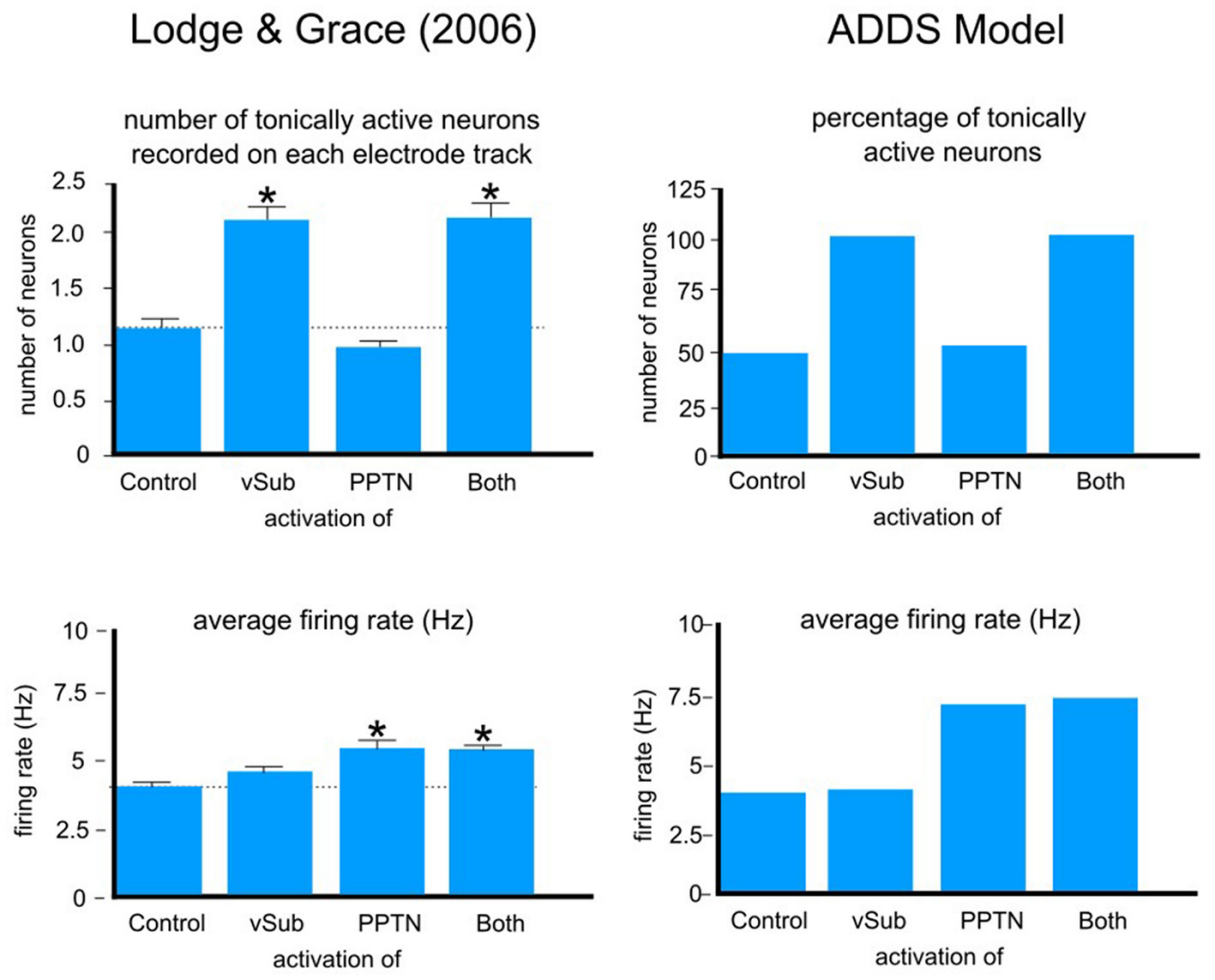
Fits of a computational version of ADDS to the data of Lodge and Grace (2006). vSub, ventral subiculum; PPTN, pedunculopontine tegmental nucleus. A * signifies a statistically signficant difference from control (*p* < 0.05). Error bars indicate one standard error. Adapted from Inglis et al. (2021).

Using this computational version of the model, Inglis et al. (2021) then showed that it provides excellent quantitative fits to a variety of relevant neuroscience results.^1^ One example is shown in Figure 2. The left column shows data reported by Lodge and Grace (2006). In this experiment, the vSub, the pedunculopontine tegmental nucleus, or both of these structures were activated in rats (via NMDA infusion), while recordings were made of the number of tonically firing VTA DA neurons and of the average firing rate of all currently active VTA DA neurons. These results were compared to results from a control condition in which neither structure was activated. The right column of Figure 2 shows predictions of the computational version of ADDS under these same conditions. Note that the model accurately predicts that vSub activation increases the number of tonically active VTA DA neurons without increasing the firing rate of active neurons, whereas activation of the pedunculopontine tegmental nucleus has the opposite profile—that is, the firing rate of active DA neurons increases but the number of tonically active neurons remains the same. In ADDS, increases in the sense of agency increase vSub output, so Figure 2 shows that ADDS predicts that increases in agency should increase the number of tonically active DA neurons in the VTA, but not increase the firing rate of those DA neurons that are tonically active. In the Figure 1 model, an unexpected reward will increase activity in the pedunculopontine tegmental nucleus, which will cause phasic firing of tonically active DA neurons. As a result, ADDS predicts that agency and reward have independent effects on the DA system. Changes in agency change the number of tonically active DA neurons but do not cause phasic firing, whereas unexpected reward causes phasic firing but does not change the number of DA neurons firing tonically. The good fits shown in Figure 2 and the other results reported by Inglis et al. (2021) support the validity of the specific neural network illustrated in Figure 1, which was used to derive all of the behavioral predictions of ADDS.

The ADDS model, as described in Figure 1 and by its computational version, greatly oversimplifies the neuroanatomy and neurophysiology of the brain regions depicted there. For example, virtually all of these regions include multiple neuron types (e.g., projection neurons, as well as interneurons) that have different tonic firing rates, and different resting membrane potentials. Nevertheless, simplified models of this type can be useful if they account for data and suggest new experiments because they establish a minimal set of anatomically-supported connections that are sufficient to account for the phenomena under study. For example, Figure 2 establishes that the good fits shown there do not require a model that includes interneurons. This does not mean, of course, that interneurons do not contribute to nucleus accumbens or ventral pallidal dynamics. But it does mean that a simplified model, like ADDS, can nevertheless mimic network-level dynamics well enough to make extensions of the theory to behaviors of the type considered in this article a reasonable endeavor.

Dopamine affects many behaviors (for reviews, see, e.g., Ashby et al., 2015, 2024) and dysfunction in DA neurotransmission is associated with a wide variety of neuropsychiatric disorders, including Parkinson's disease, schizophrenia, and depression (e.g., Grace, 2016). As a result, it is no surprise that ADDS makes many novel and testable behavioral predictions. Many of these were derived by Ashby et al. (2024), who also showed that each of these predictions has strong preliminary empirical support (see also, Ashby and Ashby, 2026). The general approach, which is also followed in this article, was to find studies that made no mention of agency, but nevertheless showed that some behavior is affected in a certain way by an increase in brain DA levels. For example, the literature includes many studies that examined how some specified behavior is affected by drugs that either increase or decrease DA level (DA agonists or precursors in the former case, and DA antagonists in the latter case). Since ADDS predicts that increases in agency should increase DA levels and decreases in agency should have the opposite effect, it therefore predicts that changes in agency should have similar effects on the behavior as changes in DA levels caused by administering the drugs. Evidence that changes in agency have similar effects on the behavior as the drugs supports ADDS, whereas evidence that agency has dissimilar effects falsifies ADDS.

Using this approach, Ashby et al. (2024) derived many behavioral predictions from ADDS and in each case, reported promising empirical support. Describing all these predictions and their existing empirical support is well outside the scope of this article. The interested reader should consult Ashby et al. (2024) for details. Briefly though, the set of novel predictions made by ADDS includes predictions that increases in agency will (1) increase motivation, (2) improve all forms of executive function, (3) facilitate procedural learning, but only in the presence of immediate trial-by-trial feedback, (4) have little or no effect on learning-related effects due to perceptual priming or on the acquisition or expression of standard-eyeblink conditioning, (5) facilitate the development of automatic behaviors, but have little or no effect on the production of behaviors that are already automatized, (6) amplify the cognitive benefits of positive mood, and (7) reduce pain. Even more recently, Ashby and Ashby (2026) generalized ADDS by deriving novel predictions about how the sense of agency impacts all aspects of drug addiction, including (1) the acquisition and maintenance of addictive behaviors; (2) cravings, compulsions, and relapse; and (3) treatment and recovery.

## 3 Decision making under uncertainty

Every day, we make countless decisions, and not surprisingly there are rich theoretical literatures that study this process. Two largely separate theoretical approaches focus on how decisions are made in the face of uncertainty. Statistical decision theory, which is the dominant theory within the fields of psychophysics and perception, assumes that every behavior is preceded by a decision. The theory assumes that even the simple act of detecting the presence of a signal requires a decision. In statistical decision theory, which includes signal detection theory and general recognition theory, the uncertainty facing the decision maker is in the state of the world. For example, in a simple detection task, the uncertainty is about whether the current sensation is caused by a signal or by noise (i.e., in the stimulus, during sensory and perceptual processing, and/or in the memory systems that provide a long-term store for the criterion that initiates action). For a complete coverage of this approach, see Ashby (2026).

Independently of statistical decision theory, a different approach, similarly referred to as decision theory, evolved within the fields of economics and psychology. Decision theory focuses on choices that are made when the uncertainty is about the outcome that will result from an action, rather than about the current state of the environment. For many decisions, outcomes are uncertain, either because they depend on future and/or probabilistic events, or because our understanding of environmental contingencies is incomplete. The most classic and well-studied examples are gambles in which the outcome—for example, the amount won or lost—is determined by something like the toss of a coin or the roll of dice. There are two general approaches—normative and descriptive. Normative decision theory focuses on how decisions should be made if the goal is to produce the best possible outcome, whereas descriptive decision theory tries to understand how humans actually make decisions (for an overview, see, e.g., Peterson, 2017).

ADDS predicts that changes in the sense of agency should affect many behaviors, including those studied within the frameworks of both statistical decision theory and descriptive decision theory. Ashby et al. (2024) considered applications of ADDS to many tasks commonly studied within the statistical decision theory framework (e.g., tasks that recruit executive function or procedural learning), but they did not consider tasks that fall within the rubric of decision theory. Therefore, this article focuses on the predictions that ADDS makes about decisions made when the outcomes are uncertain—that is, about tasks commonly studied within descriptive decision theory.

One of the oldest and still most influential descriptive-decision theories, which dates back to Daniel Bernoulli in 1738 (reprinted as Bernoulli, 2011), assumes that people choose the option with the greatest subjective value or utility. Modern versions of this proposal assume that subjective value, or utility, depends on a variety of factors, including objective value, subjective reward probability, beliefs about the environment, risk, and the temporal delays associated with the various possible outcomes. For example, these assumptions are shared by subjective-expected utility theory (Edwards, 1954) and by prospect theory (Kahneman and Tversky, 1979).

According to these accounts, sense of agency could influence how decisions are made if it affects any of these component processes. The next four sections consider predictions of ADDS about how changes in the sense of agency affect decisions that are made when outcomes are uncertain by affecting (1) how those outcomes are valued (e.g., their utility), (2) the decision maker's willingness to task risk, (3) the decision maker's estimate of the probability that the various possible outcomes occur, and (4) how delays in those outcomes affect their value.

Before beginning, however, it is important to note that changes in agency are likely to affect decision making in other less domain-specific ways. For example, as noted by Ashby et al. (2024), ADDS predicts that increases in agency will increase motivation, and it seems likely that more motivated individuals are more likely to seek out environments where gains are possible. As a result, ADDS predicts that increases in agency are likely to increase decision-making behaviors in general. This is a difficult prediction to test though, because as we will see, ADDS also predicts that increases in agency increase risk-taking, and unfortunately, an increase in willingness to accept risk is often confounded with an increase in the frequency of decisions that might lead to gains. Nevertheless, there is some supporting evidence for the ADDS predictions. For example, Davis et al. (2000) reported that gamblers made more bets of all types when playing craps in a Reno casino when they rolled the dice themselves compared to when the dice were rolled by another yoked gambler. Similar results were reported by Beisswingert et al. (2016).

Similarly, ADDS predicts that increases in agency boost executive function, and therefore, another domain-general prediction of ADDS is that increases in agency should increase the rationality or optimality of any decisions that are made. Unfortunately, I know of no direct tests of this prediction, although Yavuz et al. (2025) did report evidence supporting the opposite direction of causality. Specifically, they reported that more optimal decisions—that is, decisions that led to outcomes with greater utility—were associated with a higher sense of agency than less optimal decisions.

## 4 Expected value or utility

Utility is the value that one assigns to, or the satisfaction elicited by some specific, and possibly future outcome. It is a highly subjective assessment that depends on many factors, including the objective value of the outcome, the agent's current state of need (e.g., hungry vs. sated, rich vs. poor), the probability that the outcome will occur, the agent's willingness to accept risk, and the length of the delay between the time that the choice is made and the outcome is delivered.

There is now considerable evidence that the brain DA system responds in ways that are broadly consistent with expected utility theory—at least, in the case of positive rewards. In other words, the response of the DA system to outcomes and to cues that predict outcomes seems to be affected in the same way and by the same factors as utility. First, ordinal rankings of DA responses strongly agree with ranked preferences for rewards that are collected before the DA recordings are made (Lak et al., 2014; Stauffer et al., 2016). This suggests that the DA response to a reward depends on its subjective, rather than objective value. Second, as is well known, the phasic firing of DA neurons increases with the reward prediction error (RPE), which is defined as the value of the obtained reward minus the value of the expected reward (Schultz et al., 1997). In other words, the DA response to the same reward is stronger if that reward is unexpected, and as a result, the magnitude of the DA response depends on subjective estimates of reward probability. Third, there is evidence that the DA response depends on inferences animals make about expected rewards, even when the sensory cues are ambiguous (Babayan et al., 2018; Lak et al., 2017). For example, the DA response to the same perceptual cue varies with the animal's beliefs about the magnitude of the reward the cue predicts, and does not simply reflect the cue's past reward history. Fourth, DA neurons respond to risk (Schultz, 2010). For example, DA neurons respond more strongly to a gamble with equal probabilities of two outcomes than to a sure outcome equal to the expected value of this gamble (Lak et al., 2014). Fifth, the response of DA neurons decreases with reward delay, and this temporal discounting can be described by a hyperbolic discounting function that is similar to the function that describes human behavioral temporal discounting (Day et al., 2010; Kobayashi and Schultz, 2008). Not surprisingly, there have been multiple proposals that the brain DA response to cues that predict positive outcomes operates largely as a (marginal) utility signal (Stauffer et al., 2014, 2016).

In contrast, the role that the DA system plays in decisions that involve losses is more controversial. First, it is important to distinguish between two different kinds of losses. One occurs when the outcome is aversive, which is defined here as an outcome that would elicit an avoidance response if presented in isolation. So by this definition, for example, losing money in a gamble is aversive. A different kind of loss occurs when an expected gain fails to materialize. For example, in approach-approach decisions in which all possible outcomes are gains, the smallest possible gain is typically perceived as a loss (e.g., it elicits regret). These two types of losses are fundamentally different since the former type involves an aversive stimulus or event, whereas the latter type does not.

The role of the DA system is different in these two kinds of losses. It has been known for decades that many DA neurons respond when an outcome is less favorable than expected by reducing their firing rate below baseline (Schultz et al., 1997). For many years it was thought that all DA neurons responded in this way. In fact, until about a decade ago, it was thought that there is only one homogeneous population of DA neurons in the VTA, which all have similar firing properties and differ only in the magnitude of their response (Eshel et al., 2016; Stauffer et al., 2016). More recent studies however, using newer technologies (e.g., optogenetics), have reported evidence that the VTA DA population is heterogeneous, with some DA neurons responding to value and some to the choice or action that the animal makes (Coddington and Dudman, 2019; Cox and Witten, 2019; Parker et al., 2016; Yun et al., 2020). Even the subpopulation of DA neurons that respond to value is heterogeneous, with some responding only to positive outcomes, some that decrease activity when aversive stimuli are presented, and some that increase activity to an aversive stimulus (e.g., Lopez and Lerner, 2025; Verharen et al., 2020). Furthermore, the DA subpopulations that respond to aversive stimuli project to different targets than DA neurons that respond to appetitive stimuli (e.g., they project to medial prefrontal cortex and the nucleus accumbens shell; de Jong et al., 2019; Verharen et al., 2020).

Despite the solid evidence that some DA neurons respond to aversive stimuli, there is ample evidence that the effect of DA on decisions about losses is much smaller than the effect of DA on decisions about gains. First, only a small percentage of DA neurons in the VTA appear to respond to aversive stimuli (between 3% and 14%; e.g., Mirenowicz and Schultz, 1996). Second, administering a DA precursor (levodopa) to healthy adults increases reward seeking in a gambling task that includes choices among potential gains but does not affect behavior in a task that includes choices among potential losses (Rutledge et al., 2015). Third, there is evidence that the system that responds to losses depends much more heavily on norepinephrine (NE) than on DA (Sokol-Hessner and Rutledge, 2019). For example, Rogers et al. (2004) reported that a single dose of a NE antagonist (i.e., propranolol) reduced the ability of healthy adults to discriminate between gambling losses of different magnitude when the probability of winning was low and the probability of losing was high. A similar relationship between NE and sensitivity to loss was reported in a PET-scanning study (Takahashi et al., 2013). Based in part on this evidence, Sokol-Hessner and Rutledge (2019) proposed that decisions about gains vs. losses are mediated by separate neural systems and they hypothesized that the most important projections in decisions about losses are projections from the amygdala to the striatum that are strongly modulated by NE. Using this model, they then predicted that although manipulations of DA will strongly impact decisions about gains, such manipulations will have little effect on decisions about losses. Several fMRI studies have reported evidence supporting this proposal that gain and loss prediction are mediated by separate neural systems (Knutson and Peterson, 2005; Yacubian et al., 2006; Zhang et al., 2018).

The DA system can also contribute to decisions about losses via its usual response to RPEs. However, this contribution is not straightforward. As previously noted, most DA neurons in the VTA reduce their firing below baseline when the RPE is negative and increase their firing above baseline when the RPE is positive (via the pathways shown on the right side of Figure 1). The complication is that whether an RPE is positive or negative is imperfectly related to whether the outcome was a gain or loss. For example, when a reward is received, the RPE is nevertheless negative and DA firing falls below baseline if the reward was smaller than expected (Tobler et al., 2005). Similarly, when an expected aversive stimulus fails to occur, RPE is positive and DA neurons in the VTA increase their firing above baseline (Salinas-Hernández et al., 2018). So whether an RPE is positive or negative, and therefore whether DA firing increases above or falls below baseline is not a reliable signal of whether a gain or loss occurred.

In summary, although DA seems critical when choosing among options expected to have positive outcomes, DA appears to play a much more minor role in decisions in which losses are likely. This is not to say that DA plays no role in loss decisions. The evidence suggests that it contributes in two different ways. First, there is a relatively small DA subpopulation that responds to aversive stimuli and these neurons project to regions that seem to participate in loss decisions, including for example, the amygdala and the medial prefrontal cortex (Yacubian et al., 2006; Zhang et al., 2018). Second, DA could affect decisions about loss because many losses will generate negative RPEs. For these reasons, increases or decreases in tonic DA levels could alter decisions about loss. But if they do, the available evidence suggests that such effects should be smaller on decisions about loss than on decisions about gains. First, the subpopulation of DA neurons that responds to aversive stimuli is small. Second, whether an RPE is positive or negative is imperfectly related to whether the outcome was a gain or a loss. Third, considerable evidence suggests that decisions about losses are mediated by a neural network that is mostly separate from the network that mediates decisions about gains. And fourth, this separate network seems to be affected more by NE than by DA.

**Figure 3:**
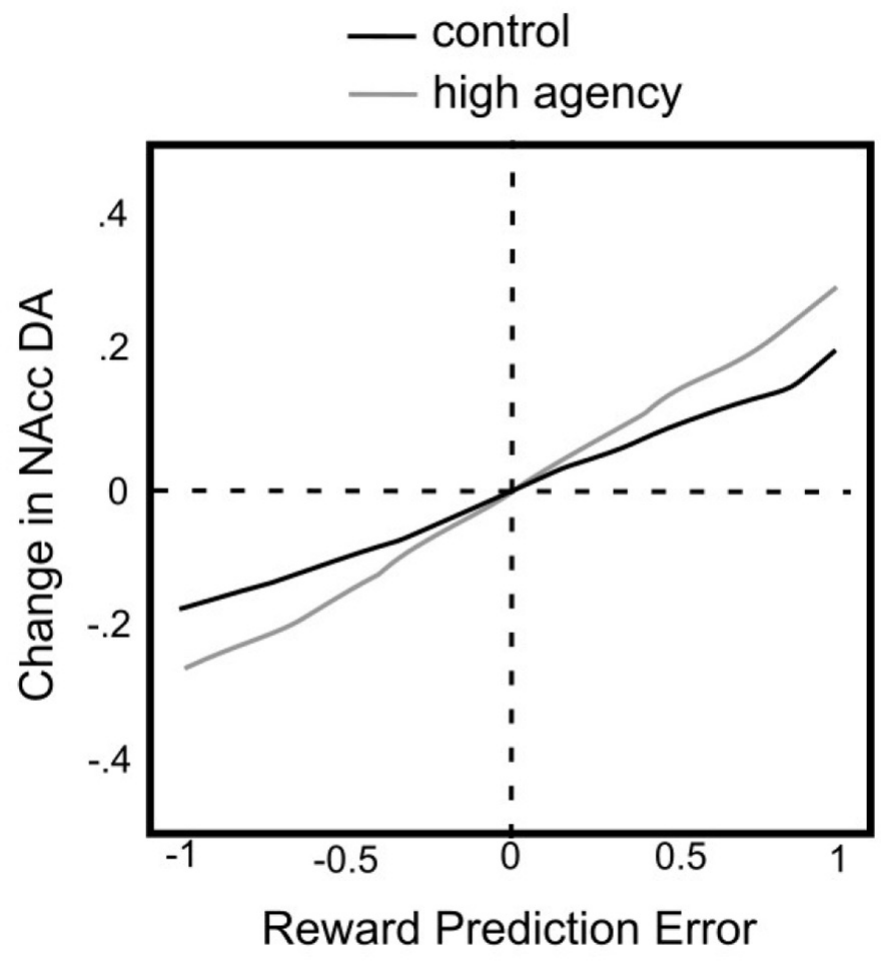
Changes predicted by the computational version of ADDS in extracellular DA concentration in the NAcc as a function of RPE, under control conditions (black curve) and when agency is high (gray curve). Adapted from Inglis et al. (2021).

What does ADDS predict? ADDS predicts that an increase in agency will increase the number of tonically active VTA DA neurons, but how does the theory get from this prediction to predictions about utility? The key variable is RPE. ADDS assumes that tonically active DA neurons in the VTA respond to RPE, with phasic bursts for positive errors (because of excitatory input from the PPTN) and suppression below baseline for negative errors (because of inhibatory input from the RMTN). Changes in agency affect these predictions because they affect how many tonically active DA neurons participate in these responses. Quantitative predictions of ADDS are shown in Figure 3. These were derived by Inglis et al. (2021) from the computational version of the the model under two conditions—one where the output of vSub is weaker and one where that output is stronger. The figure shows the changes the model predicts in the concentration of extracellular DA in the nucleus accumbens as the RPE changes. Note that the model predicts that an increase in agency should steepen these curves. Prospect theory assumes the value of an outcome is judged relative to a reference point, which is the neutral or expected outcome, rather than to any absolute value (Kahneman and Tversky, 1979). Prospect theory is flexible and places only minimal constraints on the reference point. Even so, a natural interpretation would be to assume that the reference point of prospect theory is equivalent to an RPE of zero. In this case, the zero point in Figure 3 would map to the zero point of a utility curve. The ordinate in Figure 3 is change in DA concentration. The results reviewed earlier in this section suggest that, in the case of gains, this change should be monotonically related to utility, but there is no reason to expect this relationship to be linear. ADDS makes no strong predictions about the exact shape of utility curves. But it does make the strong prediction that increases in the sense of agency should increase the utility of rewards. And the evidence that DA plays a much more minor role in decisions about losses leads to the further prediction that an increase in agency should cause the increase in the utility of gains to be greater than any possible change in the utility for losses.

**Figure 4:**
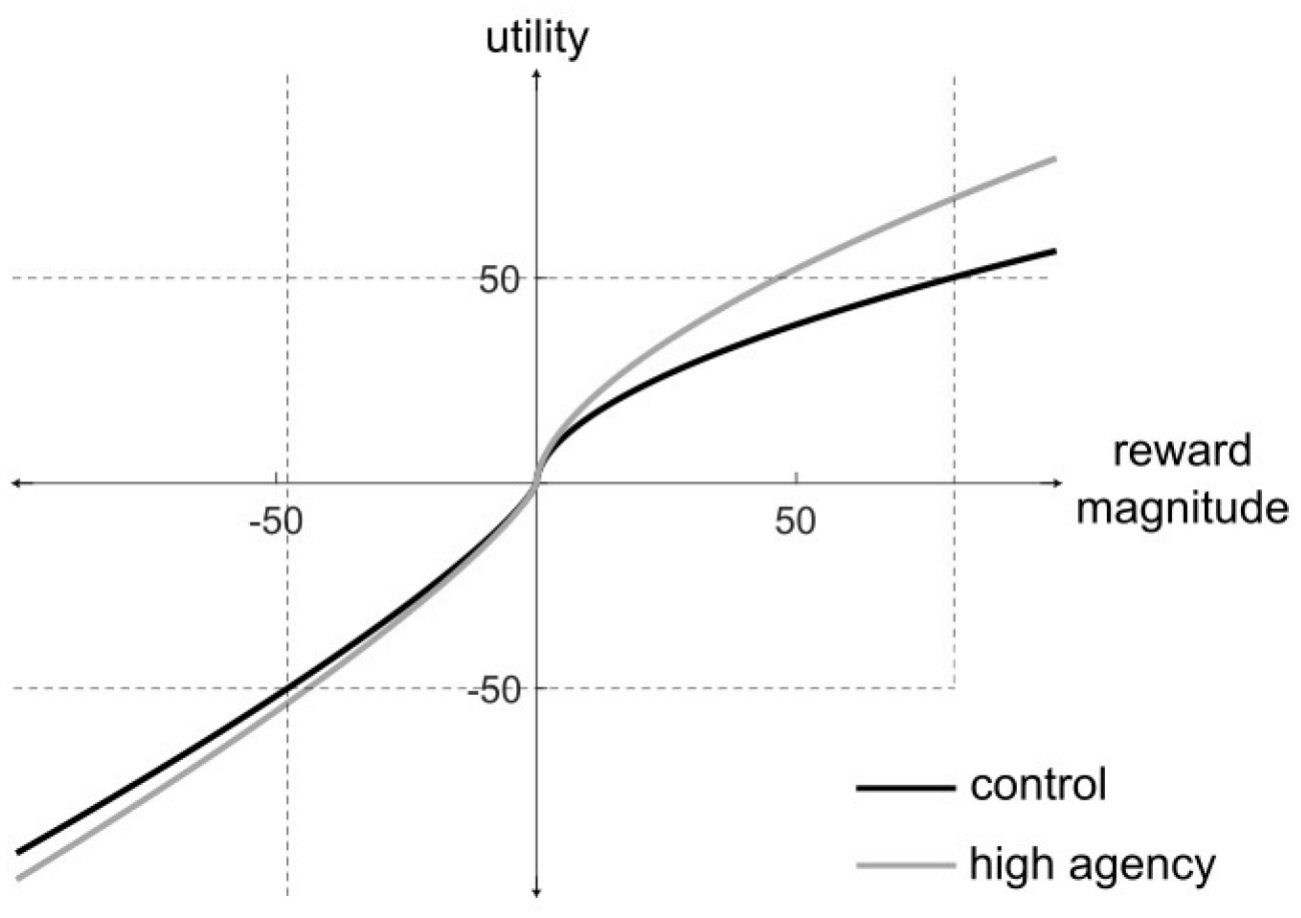
Two hypothetical utility curves in which an increase in agency affects the utility of gains more than the utility of losses.

This prediction is illustrated in Figure 4, which shows two hypothetical utility curves—one under normal, control conditions, and one under conditions in which sense of agency has increased. Note that ADDS makes two strong predictions here. First, when agency increases, the same reward should feel larger and/or more valuable. And second, this effect should be substantially weaker, and/or qualitatively different for losses. The remainder of this section considers these two predictions in turn.

Many studies have reported evidence that strongly supports the ADDS prediction that increases in agency amplify the utility of rewards. A number of different experimental paradigms have been used, as well as a variety of different participant populations, including for example, healthy human adults, children, various neuropsychological patient groups, and monkeys. The typical approach, first used 75 years ago, collects some preliminary estimate of desirability for some set of objects (e.g., household items), options (e.g., vacation destinations), or food items (e.g., M&Ms). Next, participants are presented with pairs of these stimuli and are either asked to choose between the two, or else are assigned one of the two—for example, by the computer running the experiment. The experiment then concludes with a final step in which a new set of desirability estimates are obtained. The ubiquitous result is that the act of choosing a stimulus increases its desirability, and this increase does not occur if the same outcome is assigned, rather than made by active choice (e.g., Brehm, 1956; Cockburn et al., 2014; Egan et al., 2007; Sharot et al., 2010). All theories of agency predict that active choice increases agency, so these studies all support the ADDS prediction that increases in agency increase the utility of rewards.

Many earlier articles demonstrating these effects speculated that they might be due to some form of cognitive dissonance. The idea was that the participant might go through some thinking like “I earlier chose A over B and I am a rational being, therefore A must be more valuable than B.” However, Cockburn et al. (2014) showed that the effect is at least partly due to brain DA levels, by showing that the magnitude of the choice bias—that is, the amount that free choice increases the value or utility of the outcome—is larger in individuals with a DA-related gene that increases the efficacy of available DA.^2^ Supporting evidence for the role of DA and the reward system in this choice bias was provided by Leotti and Delgado (2011), who ran the choice part of this task inside an fMRI scanner. They reported that active choice activated the ventral striatum more than when the same reward was assigned and similar effects were found in other regions known to participate in reward processing (e.g., dorsal anterior cingulate). Even stronger evidence comes from Rutledge et al. (2015), who reported that the DA precursor L-DOPA increased reported happiness when the same small reward was received in a gambling task. All of these results support the ADDS prediction that increases in agency increase the utility of positive outcomes.

In contrast to the literature on how active choice affects the utility of gains, far fewer studies have examined how active choice affects the utility of losses. The most direct test of the ADDS prediction that active choice has less effect on the utility of losses than gains was reported by Leotti and Delgado (2014). In this experiment, participants completed separate blocks in which they had to choose between two visual cues associated with two possible gains or two possible losses. In one condition, participants chose the cue that they thought would generate the greatest gain or the least loss, whereas in another condition, this choice was made by the computer. At the end of the experiment, participants rated how much they liked or disliked each of the visual cues. In the gains condition, participants liked the cues they selected more than the cues that the computer selected, which replicates results reviewed above and supports the ADDS prediction that active choice increases the utility of gains. In the losses condition, however, there was no difference in the (dis)liking scores for cues that were actively chosen vs. cues that were chosen by the computer. Note that this result contradicts the cognitive dissonance hypothesis, since cognitive dissonance should be the same on gain vs. loss trials. In summary, these results support the ADDS prediction that active choice has less effect on the utility of losses than gains. Even so, much more work is needed to test this prediction rigorously.

It is also important to note that none of the studies I cited as evidence that utility and DA are closely related mention agency, nor do any of the studies showing that active choice increases utility. Furthermore, none of the results described in any of these studies imply or even suggest that active choice increases utility because it increases agency. ADDS makes the strong and novel prediction that sense of agency ties the disparate DA and active-choice literatures together. Significantly, note that ADDS strongly disagrees with the predominant theoretical view that active choice increases utility because of cognitive dissonance. For these reasons, the prediction that active choice should increase the utility of gains and have a smaller effect on the utility of losses is novel to ADDS and not a circular recasting of results already in the literature. As evidence of this, note that ADDS makes novel predictions that are untested in the literature. For example, I know of no studies that have tested the ADDS prediction that the DA response should increase when an outcome occurs because of active rather than passive choice. Similarly, I am unaware of any studies that have tested the ADDS prediction that an increase in agency by some means other than active choice should also increase utility.

It is true that some of the studies I cited to justify the ADDS predictions about how agency affects utility included conditions in which an animal made an active choice. For example, Lak et al. (2014) had monkeys choose between outcomes that differed in reward magnitude and risk. A human making such choices would likely have higher agency than immediately before the choice period began. Even so, note that the ADDS predictions summarized in Figure 4 do not follow from this observation because none of those studies compared the DA response to active vs. passive choice, nor to any two conditions that would likely cause agency to differ. To my knowledge, no one has compared the results of active choice to results of a condition in which monkeys are assigned the same outcomes with no opportunity for active choice, nor has anyone previously speculated or predicted that the DA response to active choice would be different if the same outcome was assigned without choice.

## 5 Risk taking

ADDS makes another strong utility-related prediction about decision making. The vertical dotted lines in Figure 4 indicate reward magnitudes that expected utility theory predicts should cause indifference in an equiprobable gamble under control conditions. Specifically, note that for the black utility curve, a gain of 79 has utility +50 and a loss of –48 has utility –50. So expected utility theory predicts that this hypothetical participant would be indifferent to a gamble in which a coin toss yields of win of 79 (e.g., dollars) on heads and a loss of 48 on tails. But note that under high agency conditions, the participant would now strongly favor this gamble because a gain of 79 now has utility +69, whereas a loss of 48 now has utility –53. So the expected utility of the gamble when agency is high is now


$$\begin{array}{l} EU=0.5\left(69\right)+0.5\left(-53\right)=+8. \end{array}$$ (1)

Note that because of the ADDS prediction that an increase in agency affects positive utilities more than negative utilities, there are now gambles that would be accepted when agency is high, but rejected when agency is lower. As a result, a participant described by the gray utility curve in Figure 4 would be described as more risk seeking than a participant described by the black utility curve. In other words, ADDS predicts that increases in agency should increase risk-taking behaviors—primarily because the potential rewards expected from success are now more highly valued, and because there is not an equal but opposite concomitant change in the utility of possible losses.

Direct evidence supporting this ADDS prediction comes from a number of studies that used the Balloon Analog Risk Task (BART; Lejuez et al., 2002) to study how agency affects risk taking (Chen and Zhang, 2025; Damen, 2019; Karsh et al., 2021). In this task, participants are shown a picture of a virtual balloon along with a button that if clicked, will pump air into the balloon. The participant's task is to inflate the balloon and each click of the pump earns a reward. However, if too much air is pumped into the balloon, it will pop, in which case all rewards are lost. Agency was manipulated in two ways. One method varied the delay between the time when the pump button was clicked and the balloon increased in size (Damen, 2019; Karsh et al., 2021). In the high-agency conditions, this effect was immediate, whereas in the low-agency conditions there was a half-second delay. Previous research has validated that varying the time between an action and its outcome is highly effective at manipulating sense of agency (Sato and Yasuda, 2005). A different approach was to vary the participant's control of the pump (Chen and Zhang, 2025). In the high-agency conditions, the participant had full control of the pump, whereas in the low-agency conditions, the pump was controlled by the computer and the participant was mostly just an observer. All theories of agency predict that agency is higher when an outcome is produced by one's own actions, rather than by some external force or agent. All of these studies reported that, as predicted by ADDS, participants took more risks when agency was high.^3^

ADDS predicts that increases in agency should increase risk taking and that this effect is mediated primarily by the brain DA system. Although the BART studies directly tested whether increases in agency increase risk taking—and these studies all support the ADDS prediction—many more studies have reported that increases in brain DA levels increase risk taking. So these studies all indirectly support ADDS. Specifically, many studies on healthy adults, various neuropsychological patient groups (e.g., Parkinson's disease patients), and nonhuman animals have reported that drugs that increase DA neurotransmission (DA agonists or precursors) increase risk-taking behaviors (e.g., Cools et al., 2003; Gross et al., 2021; Riba et al., 2008; Rigoli et al., 2016; Rutledge et al., 2015; St Onge and Floresco, 2009).^4^ One well-documented example of this occurs when the symptoms of Parkinson's disease are treated with DA replacement therapies. Many studies have noted that a prominent side effect of these treatments is a possible increase in risky behaviors, such as compulsive gambling, hypersexuality, and binge eating (e.g., Baig et al., 2019; Hassan et al., 2011). Other supporting evidence was reported by Chew et al. (2019), who used real-time fMRI to show that the propensity to accept a risky gamble changes moment-to-moment with real-time fluctuations in DA neural activity. Similarly, Freels et al. (2020) reported a positive correlation between phasic DA release in the nucleus accumbens (shell) and risk taking in rats.

There is also evidence that risk taking in the BART is modulated by DA. First, Claassen et al. (2011) reported that Parkinson's patients took more risks in the BART when they were on DA replacement medication than when they were off these medications. Second, Kohno et al. (2015) similarly concluded that DA modulates risk taking in the BART—a conclusion they reached by analyzing results from a large fMRI study of the BART, and from a subsequent PET-scanning study that measured binding potential at DA D2 and D3 receptors.

Furthermore, there have even been proposals that DA increases risk taking in exactly the manner predicted by ADDS. Specifically, Rigoli et al. (2016) hypothesized that DA may increase risk taking because it “modulates the attractiveness of surprising outcomes” (p. 2665)—a conclusion that agrees perfectly with the account proposed here.

In summary, there is substantial evidence supporting the ADDS prediction that increases in agency increase risk taking. Furthermore, there is also strong evidence that increases in brain DA levels increase risk taking. ADDS links these two proposals in a causal way, and provides a neuroscientific account of these results.

## 6 Subjective probability

Many theories of decision making assume that decisions are made by choosing the option that has the greatest sum


$$\begin{array}{l} U=\overset{r}{\underset{i=1}{\sum }}\pi \left(i\right)u\left(i\right), \end{array}$$ (2)

where *U* is expected utility or value, *u*(*i*) is the utility or value associated with the *i*th of the *r* possible outcomes that can occur if this choice is made and π(*i*) is an estimate of the likelihood or probability that outcome *i* occurs, or else is a number that increases with that probability. In subjective-expected utility theory, π(*i*) is a subjective estimate of the true probability that outcome *i* occurs, and is a true probability in the sense that the π(*i*) are constrained to add to 1 (Edwards, 1954). In prospect theory (Kahneman and Tversky, 1979) and in some extensions of expected utility theory (Schmeidler, 1989), the π(*i*) are subjective weights that increase monotonically with probability, but they are not themselves probabilities because they are not constrained to add to 1. The more important point here is that all of these theories predict that decisions are based on judgments of the probabilities that the various possible outcomes occur. So another way that agency could affect decision making is by changing the subjective probabilities or weights—that is, by changing the values of π(*i*). Compared to work on utility, there is relatively little research that allows strong predictions to be derived from ADDS about how changes in agency might affect these estimates. Even so, there are a few results worth noting.

It has been known at least since the seminal work of Kahneman and Tversky (1979) that people almost universally overestimate small probabilities and underestimate large probabilities. Several studies examined the role that DA might play in these biases. First, using PET scanning, Takahashi et al. (2010) reported that human participants with less binding of DA to D1 receptors in the striatum had more exaggerated biases than participants with more D1-receptor binding. Second, several studies reported that DA receptor D2 antagonists have the opposite effect—that is they reduce both biases (Burke et al., 2018; Ojala et al., 2018). In other words, when there was less activation of DA D2 receptors, people overestimated small probabilities and underestimated large probabilities by a lesser amount. These results all suggest that the amount by which people overestimate small probabilities and underestimate large probabilities is modulated by DA. As a result, ADDS predicts that changes in the sense of agency are likely to affect how probability is estimated or weighted. Even so, much more work is required to sharpen this prediction. In particular, a better understanding is needed of exactly how increases and decreases in the number of tonically active VTA DA neurons affects subjective estimates of probability.

## 7 Temporal discounting

In typical laboratory studies of decision making, participants are given choices between gambles with different possible immediate outcomes. In contrast, many real-life decisions are among actions that have outcomes that occur at different points of time—for example, deciding whether to splurge at a Michelin-starred restaurant tonight or save the money that would be spent there for a vacation next summer. Such decisions require considering how delays affect the value of a future reward. The decrease in value of a delayed reward is called temporal discounting.

For example, consider a reward that if given immediately would have utility *U*. Then the standard temporal discounting model assumes that if the reward is delayed by an amount of time equal to *t* then its current utility equals *U* × *D*(*t*), where *D*(*t*) is the discounting factor that is constrained to equal 1 when *t* = 0 and *D*(*t*) ≤ 1 for all *t* > 0. For any given time *t*, the smaller the value of *D*(*t*), the greater the temporal discounting. A popular model assumes *D*(*t*) has the hyperbolic form (Mazur, 2013).


$$\begin{array}{l} D\left(t\right)=\frac{1}{1+\alpha t}, \end{array}$$ (3)

where α > 0 is a constant that increases with the amount of temporal discounting. Temporal discounting amounts vary considerably across time and across individuals. For example, compared to healthy adults, it is well known that temporal discounting is greater in children (e.g., Steinberg et al., 2009) and in substance-use disorders (Bickel et al., 2014).

DA neurons also temporally discount future rewards—that is, they respond more weakly to a cue that predicts a delayed reward than to a cue that predicts immediate delivery of the same reward (Day et al., 2010; Kobayashi and Schultz, 2008). Furthermore, Kobayashi and Schultz (2008) showed that this reduction in the magnitude of the DA response with delay is well described by the Equation 3 hyperbolic discounting model. Specifically, they showed that Equation 3 provided good fits to their data and that it fit better than a model that assumed an exponential decay. So the temporal discounting displayed by DA neurons has the same qualitative form as the temporal discounting of human choice behavior.

Of course, the observation that DA neuron firing displays hyperbolic temporal discounting does not logically imply that the temporal discounting that is ubiquitous in human choice behavior is driven by the DA system. Not surprisingly though, this hypothesis has been a focus of research for many years (e.g., Koffarnus et al., 2011). However, early studies were inconsistent, with some reporting that DA increases increased temporal discounting, others reporting that it decreased discounting, and still others reporting no effect of DA. Smith et al. (2025) hypothesized that these inconsistent results occurred for a variety of reasons, including small sample sizes, few trials of behavioral data, differences in the methods used to stimulate the DA system, and because the effects of DA on temporal discounting might not be monotonic. Specifically, Smith et al. (2025) proposed that the relationship between DA levels and temporal discounting might be U shaped—that is, when DA levels are low, a modest increase in DA reduces temporal discounting, but when DA levels are high, the opposite occurs. According to this hypothesis, delivering a DA agonist to a participant with low endogenous DA levels will reduce temporal discounting, whereas delivering exactly the same drug to a participant with high endogenous DA levels will increase temporal discounting—in fact, it will increase impulsivity in general. This non-monoticity hypothesis is highly reasonable because the evidence is good that there is a similar non-monotonic relationship between DA levels and executive function—specifically, DA improves executive function up to a point, but when DA levels are high, then further increases in DA become detrimental (Ashby et al., 1999; Cools and D'Esposito, 2011).

When these weaknesses are corrected, for example with large sample sizes, administering the DA precursor L-DOPA to healthy adults reduces temporal discounting, relative to a placebo (Smith et al., 2025). A recent meta-analysis confirmed this conclusion (Sarmiento et al., 2023), noting for example, that Parkinson's disease patients, who have reduced brain DA levels, temporally discount more than healthy controls (Pennisi et al., 2023).

Based on these results, ADDS makes another strong decision-making prediction—namely, that increases in agency should reduce temporal discounting, except perhaps in cases in which brain DA levels are already extremely high, in which case increases in agency could cause impulsivity. A number of studies have directly tested this prediction, and in each case, the results support this ADDS prediction.

Gneezy et al. (2020) reported the results of two experiments that directly tested whether an increase in sense of agency affects temporal discounting. In both studies, participants first completed an irrelevant task and then made decisions about whether they preferred an immediate smaller reward or a later larger reward. Agency was manipulated by providing participants in a high-agency condition with the choice to control some stressful aspect of the irrelevant task. In one experiment they could choose to receive more time to complete the task and in the other experiment they could choose to eliminate a loud distracting noise. Critically, in both experiments these choices incurred a significant cost, and because of this, almost all participants chose not to exercise their choice. As a result, almost all participants in both conditions completed the irrelevant task under identical conditions. Nevertheless, in both experiments, the groups given the option to choose—that is, the groups with higher agency—displayed less temporal discounting because they chose larger delayed rewards significantly more often than the group without any choice.

A number of other experiments provide indirect support for the ADDS prediction that increases in agency should reduce temporal discounting. Sense of agency was not a primary focus of these studies, but they nevertheless manipulated some variable that likely affected the sense of agency of their participants, and then reported that temporal discounting was reduced in the condition in which agency was likely higher.

Many of these studies used an intervention called episodic future thinking, in which the participant vividly imagines themselves pre-experiencing a future event (Atance and O'Neill, 2001). Numerous studies have reported that episodic future thinking decreases temporal discounting (Bromberg et al., 2017; Peters and Büchel, 2010; Ye et al., 2022). Although I know of no direct tests of whether this intervention increases agency, there are several reasons to believe that it does. First, participants choose the event for imagining and as already noted, active choice increases agency (e.g., Bandura, 1977). Second, the imagery that is solicited is typically of the participant executing some action. For example, the participant might vividly imagine walking on a Hawaiian beach on an upcoming vacation, or attending the wedding of a close friend. By definition, acting on the environment increases agency. Third, there is evidence that visual imagery of the type encouraged during the induction of episodic future thinking increases agency (Gupta et al., 2025; Silvanto, 2025). So overall, studies showing that episodic future thinking reduces temporal discounting provide indirect support for ADDS.

Other evidence supporting the ADDS prediction that increases in agency should reduce temporal discoutning comes from a large study of how spatial representations affect how time is perceived. In this study, 599 participants completed a series of tasks (e.g., map reading) that included making choices between smaller immediate rewards or larger delayed rewards (Fletcher et al., 2024). Although sense of agency was not a focus of this study, across all conditions, participants who reported greater perceived control over the period of time until the future reward would be received engaged in less temporal discounting.

Unfortunately, I know of no studies that provide a direct test of the prediction that an increase in agency in a participant with already high endogenous DA levels will increase temporal discounting. Even so, there is considerable indirect evidence that, at the very least, makes this a reasonable prediction. For example, a well-documented side effect of DA replacement therapy as treatment for symptoms of Parkinson's disease is an increase in impulse control disorders (for reviews, see e.g., De Wit et al., 2022; Grall-Bronnec et al., 2018). Furthermore, several studies have reported that this increase in impulsivity includes an increase in temporal discounting (Milenkova et al., 2011; Voon et al., 2010), although at least one other study reported the opposite result—namely that medicated Parkinson's disease patients show less temporal discounting than unmedicated patients (Foerde et al., 2016). At first glance though, reports that DA replacement therapy increases impulsivity seem to disconfirm the ADDS prediction since Parkinson's disease is caused by the death of DA neurons and therefore is characterized by abnormally low brain DA levels. For this reason, it appears that ADDS might predict that DA replacement therapy should reduce impulsivity and temporal discounting in Parkinson's disease patients, not cause increases. It is important to note however, that DA replacement therapy is prescribed primarily to alleviate the motor deficits associated with the disease, typically without regard to any possible effects the medication may have on cognition (e.g., Pringsheim et al., 2021). As it happens, Parkinson's disease does not cause an equal loss of DA in all brain regions. Rather it is a progressive disease that tends to cause motor deficits before cognitive deficits (e.g., Mack and Marsh, 2017). Furthermore, there is considerable individual difference with respect to which brain regions are most impacted (e.g., Fraza et al., 2025). These observations led to the DA overdose hypothesis, which proposes that DA replacement therapies that alleviate motor deficits caused by Parkinson's disease can, at the same time, cause deficits in cognitive tasks that are mediated by cortical regions that have not yet been significantly affected by the disease (Vaillancourt et al., 2013). The idea is that the DA replacement therapy causes an overdose of DA in as-yet-unaffected brain regions where DA levels are not yet significantly reduced. This pushes the DA level in these areas past the bottom of the U-shaped curve that describes the effects of DA on temporal discounting, and as a result, for many Parkinson's disease patients, DA replacement therapy increases impulsivity and temporal discounting. Note that the DA overdose hypothesis also provides one possible account of why different Parkinson's disease studies might disagree about whether DA replacement therapy increases or decreases temporal discounting, especially when sample sizes are small. Specifically, studies that over sample patients with damage limited to motor areas are likely to report that medication increases temporal discounting (because of overdosing in unaffected cortical areas that mediate decision making), whereas studies that over sample patients with damage that extends to cortical areas are likely to report the opposite result.

## 8 Discussion

ADDS predicts that increases in agency increase the number of tonically active DA neurons in the VTA. Such an increase raises tonic DA levels in all targets of VTA DA neurons and increases the gain on the DA response to any given RPE. Because of these changes, ADDS therefore predicts that agency could affect any behavior that depends on DA. Previous work used this theory to derive predictions about how changes in agency affect (1) many different behaviors, including executive functions, skills that are acquired via procedural learning, perceptual priming, eyeblink conditioning, and automatic behaviors; (2) key psychological states, including motivation, positive mood, and pain (Ashby et al., 2024); and more recently, (3) substance-use disorders (Ashby and Ashby, 2026).

This article extended ADDS to decision making by considering how agency affects various components of the decision-making process, including utility, risk, subjective probability, and temporal discounting. The most important conclusions were that: (1) increases in agency should increase the value of positive outcomes, but have relatively little effect on the value of negative outcomes; (2) these asymmetric changes in utility should increase risk taking; and (3) increases in agency should reduce temporal discounting, except in cases where endogenous DA levels are unusually high, in which case increases in agency should increase temporal discounting. Although much more empirical work is needed to test these predictions rigorously, considerable existing evidence supports each of these predictions.

An obvious and interesting follow-up question is whether these predicted effects have adaptive value. Of course, any answer to this question must be speculative, but even so, there do appear to be some obvious adaptive advantages. Consider first the prediction that risk taking increases with agency. As mentioned earlier, agency is the sense that one has control over one's own actions and the consequences that result from those actions (Moore, 2016). In an environment where one has little control over the consequences of one's actions—that is, where agency is low—a risk-averse strategy seems wise. Executing a risky behavior when one has no confidence in the outcome of that behavior seems foolish. As the outcomes of our behaviors become more predictable and agency increases, then executing a risky behavior that might deliver a large reward starts making more sense.

A similar logic explains the adaptive benefit of reducing temporal discounting as agency increases. In an environment where the outcome of our behavior is unpredictable, an immediate small reward that is guaranteed might be preferable to a larger delayed reward because the delay in an unpredictable environment might make the delayed reward seem unpredictable. This logic seems to fit perfectly the proverbial saying “a bird in the hand is worth two in the bush.” As the outcome of our behaviors become more predictable, then the future reward begins to seem more certain, in which case a rational strategy is to wait for the larger reward.

This article focused exclusively on how changes in the sense of agency affect DA neurons in the VTA. It is important to note, however, that the midbrain includes two prominent clusters of DA neurons—one in the VTA and one in the substantia nigra pars compacta (SNpc). The SNpc DA neurons project primarily to the basal ganglia and motor-related brain regions, forming the so-called nigrostriatal DA system. Many DA neurons in both regions respond to RPE (e.g., Bayer and Glimcher, 2005), but there are key differences in their response properties. A complete review is beyond the scope of the present article, but briefly, VTA DA neurons respond more to value, whereas SNpc DA neurons are more tuned to the choice or motor response an animal is about to make (e.g., Costa and Schoenbaum, 2022; Fraser et al., 2023; Lerner et al., 2021). The types of decision making considered in this article depend on executive function and value-based reasoning, which are mediated principally by limbic areas and frontal cortex—all of which receive their DA almost exclusively from the VTA. So the focus on the VTA in the present article is appropriate. Even so, it is natural to ask what predictions ADDS makes about how changes to the sense of agency affect the activity of DA neurons in the SNpc.

Unfortunately, this is a difficult question that is beyond the scope of the current article—primarily because the relationship between the activity of DA neurons in the VTA and SNpc is complex and poorly understood. One well-known proposal is that the basal ganglia have an ascending spiral architecture that allows activity in the VTA to propagate to the SNpc (via disinhibition on successive striatal → SNpc → striatal loops; Haber et al., 2000). This account suggests that increases in agency could have similar effects on the activity of DA neurons in both the VTA and the SNpc. In support of this prediction, there is ample evidence that increases in agency facilitate procedural or motor learning that is known to depend on synaptic plasticity in striatal targets of the nigrostriatal DA system (Ashby et al., 2024). On the other hand, evidence supporting this type of spiral architectural effect is mixed. For example, some studies have reported evidence consistent with the hypothesis that the mesocorticolimbic and nigrostriatal DA systems operate mostly independently of each other (e.g., Engel et al., 2024). And Bortz and Grace (2018) reported that vSub activation by the medial septum has opposite effects on the number of tonically active DA neurons in the VTA and SNpc— increasing the number in the VTA and decreasing the number in the SNpc. Therefore, before a rigorous theory of how agency affects behaviors mediated primarily in the dorsal striatum is possible, much more work is needed on possible neural interactions between the mesocorticolimbic and nigrostriatal DA systems. In addition, behavioral experiments are badly needed that document similarities and differences in how changes in agency affect striatal-mediated vs. frontal-cortical mediated behaviors.

As this article shows, ADDS makes many highly specific and novel predictions about how sense of agency affects human behavior—predictions that would be difficult or impossible to make in the absence of a neuroscience-based theory. There is direct support for some of these predictions, and indirect support for almost all others, but a rigorous testing of the theory requires much more empirical work. Einstein and Infeld (1938) wrote that “The aim of every theory is to guide us to new facts, suggest new experiments, and lead to the discovery of new phenomena and new laws” (p. 76). ADDS certainly suggests many new experiments. Even if some future revision or extension of the theory is required to account for the results of these experiments, this new, larger database should improve our understanding of the human sense of agency.

## Data Availability

The original contributions presented in the study are included in the article/supplementary material, further inquiries can be directed to the corresponding author.

## Footnotes

1 Inglis et al. (2021) made no assumptions about the psychological construct that was modulating vSub output. Specifically, they made no mention of agency, nor did they make any attempt to account for behavioral data. The hypothesis that agency is the key driver of this network was added by Ashby et al. (2024).

2 Specifically, Cockburn et al. (2014) reported that the choice bias is larger in DARPP-32 TT carriers than in DARPP-32 C carriers.

3 Karsh et al. (2021) also reported the results of a more complex experiment in which participants first specified the number of pump clicks they wanted, and then they deflated a full balloon to a level they felt was safe for the number of clicks they had selected. Each deflating click lost money and the high agency group deflated more than the low agency group, which seems to contradict the results of the authors' earlier experiments and the results of Damen (2019) and Chen and Zhang (2025). As a test of ADDS predictions, however, more research is needed to understand this result because: (1) unlike the BART, this is not a validated method for studying risk taking; (2) as described in detail by Ashby et al. (2024), ADDS also predicts that increases in agency improve executive function and increase motivation and motor behavior, and as a result, these factors need to be disentangled from risk taking in this more complex version of the BART.

4 On the other hand, several studies have reported no effect of a nonselective DA agonist on risk taking. See Soutschek et al. (2023) for details. Petzold et al. (2019) reported that a nonselective DA agonist reduced risk taking in participants with high impulsivity, which is consistent with the hypothesis that the effects of DA on cognition are U-shaped (or inverted U-shaped), rather than monotonic. See Section 7 for details. Soutschek et al. (2020) reported that a selective D1 agonist decreased risk taking, so the increased risk taking associated with DA agonists might depend on D2 receptor activation. To my knowledge however, the Soutschek et al. (2020) result has not been replicated, so more research is needed on the relative contributions of D1 vs. D2 receptor activation to risk taking.
